# Psychiatrists as forensic authorities: evaluation of dangerous habitual offenders in West Germany during the 1960s – the Helmut Hoinka case

**DOI:** 10.3389/fpsyt.2024.1421138

**Published:** 2024-08-09

**Authors:** Oxana Kosenko, Tobias Skuban-Eiseler, Florian Steger

**Affiliations:** ^1^ Institute of the History, Philosophy and Ethics of Medicine, Ulm University, Ulm, Germany; ^2^ kbo-Isar-Amper-Klinikum, Munich, Germany

**Keywords:** preventive detention, medical opinion, forensic psychiatry, criminological prognosis, Germany

## Abstract

**Background:**

Preventive detention for highly dangerous habitual offenders has been in force in Germany for 90 years. The necessity of this measure is hotly debated from a legal perspective. However, the assignment of preventive detention is largely determined by the opinion of medical experts. This article discusses the role of medical experts and the issues they face in evaluating the dangerousness of habitual offenders using the case of the marriage swindler Helmut Hoinka, prosecuted several times in the Federal Republic of Germany in the 1960s.

**Methods:**

Helmut Hoinka’s case was chosen for analysis because of the rare opportunity to access detailed materials that allowed us to follow in detail the reasoning of the medical experts who evaluated Hoinka: medical reports stored in the Gerd Huber Archive at the University of Ulm, and Hoinka’s court case from the State Archive of North Rhine-Westphalia. To examine these sources, we implemented the historical-critical method.

**Results:**

The medical experts who evaluated Hoinka were aware of the defendant’s criminal record prior to the evaluation, which was a source of bias. In addition, the criteria for classifying the offender as a dangerous habitual offender were open to a wide range of interpretations. Hoinka’s high level of intelligence was negatively emphasized. Some test results were considered unreliable because it was assumed that Hoinka had manipulated his answers. Personal value judgments were allowed in assessing Hoinka’s personality. Hoinka’s criminal behavior was considered a medical symptom of psychopathy because it violated general moral and social norms. The medical reports of both experts showed that the psychiatrists believed in the genetic nature of psychopathy and criminal behavior. Their criminological prognosis was fully supported by the court in imposing the sentence.

**Conclusion:**

Challenges to Hoinka’s criminological prognosis were the experts’ personal biases, their belief in the theory of genetic predisposition to crime, the lack of clear criteria for antisocial personality disorder, and the absence of forensic recommendations for “psychopathic” criminals. The experts’ opinion on Hoinka’s criminal predisposition was crucial to the imposition of the sentence.

## 1 Introduction

90 years ago, on November 24, 1933, a Law against Dangerous Habitual Offenders and on Measures of Security and Correction was passed in Nazi Germany (1). That law introduced preventive detention (*Sicherungsverwahrung*) for highly dangerous habitual offenders. This category included the following criminal subjects: recidivist property criminals (thieves and fraudsters), morality criminals (child molesters and homosexuals) and generally unreliable persons (beggars, vagrants, etc.). Despite the abolition of this law after World War II, preventive detention continued to be applied. In the 1960s, in the Federal Republic of Germany, the fraudsters against which preventive detention was imposed were predominantly loan scammers. More serious types of fraud, such as imposture, business formation fraud and marriage swindle, were relatively rare (2). Marriage swindle meant that the perpetrator aimed from the very beginning to enrich himself under pretense of an intention to marry which, in reality, was non-existent. The reform of criminal law in 1969 did not lead to the abolition of the controversial preventive detention (3). The main critic point of the legal experts was that a “post-punishment punishment” with an open duration of detention is a violation of human rights (4). The regulation of preventive detention was reformed in 2013, but still remains in German criminal law as a measure of correction and security. It still warrants criticism from experts in the field. This criticism is based primarily on notions of human rights (5, 6). Thus, the discussion is framed within a legal discourse.

However, in this paper, we would like to address the medical side of the issue. Using one prominent case of a marriage swindler as an example, we examine the role of psychiatrists whose responsibility is not only to assess the sanity of criminals, but also their potential for reoffending, and to identify possibilities of medical therapy and resocialization. The analysis of this case will allow us to identify the challenges psychiatrists encounter in forming an opinion on dangerous offenders and assessing their potential risk to society in the future. For this purpose, we structured our paper as follows. Firstly, we introduce the case of Hoinka and the reports of the two medical experts prepared for the court between 1964 and 1965. We also outline the fate of the marriage swindler after the trial to trace the need for preventive detention in his case. Secondly, in the discussion, we analyze the opinions of both experts in terms of biases, criteria for diagnosing a psychiatric disease like a personality disorder, argumentation regarding the therapy, and prescription of preventive detention. This will allow us to identify and discuss the challenges psychiatrists faced in evaluating the dangerousness of the offender.

**Figure 1:**
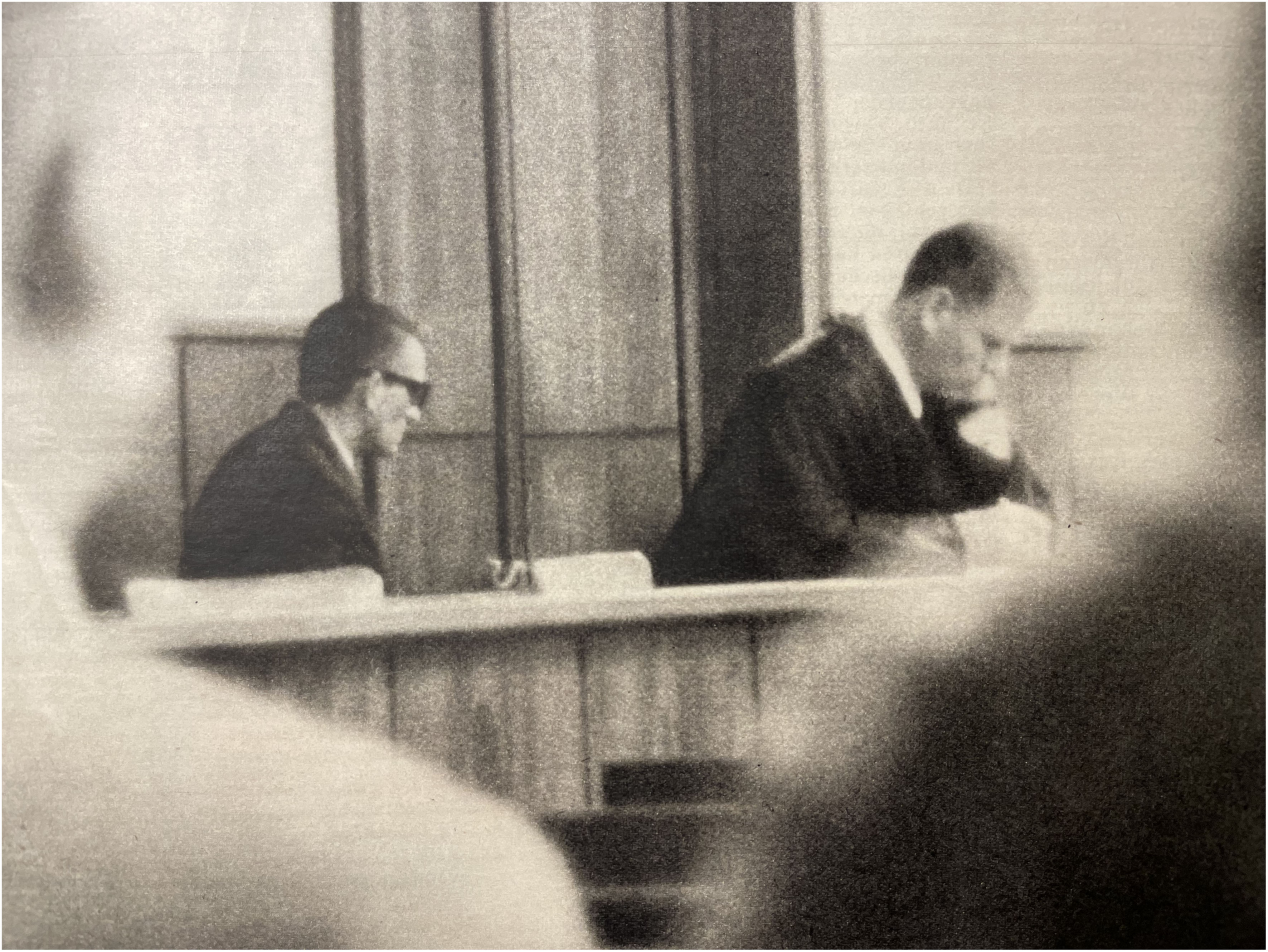
Helmut Hoinka (on the left) during the hearings in 1965. Reproduced from: Rosen ziehen immer. Warum so viele Frauen auf Heiratsschwindler Hoinka hereingefallen sind. *Quick* (1965) 39(18):100. Public domain.

In July 1965, the newspapers of the Federal Republic of Germany followed with astonishment the public trial of the marriage swindler Helmut Hoinka (**Figure 1**). Journalists wondered how a fifty-year-old man of ordinary appearance, with no education and no outstanding intellectual abilities managed to deceive dozens of women and defraud them of approximately 200,000 Deutsche Mark, which today would amount to about half a million euros. The affected women were ashamed to admit that they were victims of deception by a skillful gentleman, who never failed to bring a bouquet of scarlet roses to a date. For that reason, the press nicknamed him “The Rose-Bearer”. However, the public was mainly preoccupied by how women in future could be protected from that highly dangerous marriage swindler? The question of how to deal with recidivous criminals was once again on the agenda. The greatest responsibility in this matter, however, lay not so much with lawyers as with psychiatrists. Using the Hoinka case as an example, we will look at how the psychiatrists evaluated the defendant, how objective they were, on what basis they advised preventive detention, how justified the sentence was, and whether it finally proved to be meaningful. We chose this case for our analysis because we had the rare opportunity to access comprehensive materials that allowed us to follow in detail the argumentations of the medical experts who evaluated Hoinka. Moreover, since this case was widely discussed in public, the medical experts had a particular responsibility in evaluating the defendant and reasoning on the matter of preventive detention.

## 2 Materials and methods

To prepare this paper, we analyzed unpublished archival documents related to Hoinka’s trial and research works. The group of archival documents includes materials that we found in the Gerd Huber Archive, stored at the University of Ulm, and the State Archive of North Rhine-Westphalia, Rhineland Department, in Duisburg (Landesarchiv Nordrhein-Westfalen, Abteilung Rheinland). The private archive of West German psychiatrist Gerd Huber, who was one of the medical experts in the Hoinka trial, contains various documents related to the defendant’s evaluation. These documents include copies of his medical report and the report of the second medical expert, Fred Dubitscher, correspondence with the court and Hoinka’s lawyers, as well as results of the psychological examinations of Hoinka. Hoinka’s court case from the State Archive of North Rhine-Westphalia also contains important information like decisions in the criminal case against Hoinka, minutes of hearings and the sessions of the 3rd Grand Criminal Chamber of the Regional Court of Cologne.

Apart from the above-mentioned archival documents, we have also evaluated research works on the history and legal aspects of preventive detention in Germany and other countries. To examine these sources, we implemented the historical-critical method, which includes the stages of acquisition of primary sources and research works, critical evaluation of the information contained in primary sources, and presentation of historical data in historical context in terms of objectivity and significance (7).

## 3 Results

### 3.1 Helmut Hoinka’s case

Helmut Rudolf Adolf Hoinka was born in 1913. He was 51 years old at the time of his last trial in Cologne. He was born in Upper Silesia, the son of a school principal and grew up with three sisters in orderly domestic circumstances. He attended preparatory high school, where he had to repeat a class due to lack of diligence. After leaving school, he began an agricultural training program, which he abandoned in order to join the German armed forces, the Reichswehr, in 1932. He served in the Reichswehr for about three years and was discharged in 1935 for unsuitability. Before that, in 1933, he had suffered a superficial head injury during a fall from a horse. After his discharge from the Reichswehr, his father did not take him back into the parental home. Subsequently, Hoinka earned his living as a commercial agent. He did not receive any regular professional training. In 1936, he was convicted for the first time for committing fraud in connection with his activities as a commercial agent and sentenced to four months in prison. Two years later, he was sentenced once again for fraud. In 1940, his first marriage broke up after a short time. In that same year, he was sentenced to two years in a house of correction (*Zuchthaus*), a fine, and five years of loss of civil rights for repeated fraud and aggravated forgery. He served part of his sentence until 1942, before the remaining part was suspended. After that, he was drafted into the Wehrmacht. According to Hoinka, he was drafted to the artillery in Africa, and later was sent to Greece. From 1945 to 1947 he was a prisoner of war^1^.

After the end of the war, Hoinka initially worked on a small farm. In 1948, he entered a permanent employment relationship as a commercial employee, and he later tried to become self-employed. When that attempt failed, he subsequently turned more and more to earning a living through marriage swindling. In 1952, he was sentenced by the Hamburg Criminal Court to a total of two years in a house of correction for two cases of repeated recidivist fraud and for continued recidivist fraud; he had obtained of more than 8,000 Deutsche Mark by making marriage vows to nine women. Hoinka served part of this sentence until the beginning of 1954. The remainder was suspended out of mercy and remitted in 1957. After his release from the house of correction, Hoinka went to East Germany, where he initially found employment with a consumer cooperative. However, he soon resumed his activities as a marriage swindler. At the end of 1954, he was sentenced to a total of two years and six months in a house of correction for two cases of fraud. In 1955, during imprisonment, Hoinka married a second time. After serving part of this sentence, he returned to the Federal Republic of Germany in 1956, where once again he initially earned his living as a commercial agent. After a short time, however, he resurfaced as a marriage swindler and was therefore sentenced in 1957 by the Regional Court in Düsseldorf for recidivist fraud and for attempted recidivist fraud as a dangerous habitual criminal to a total sentence of two years and six months in a house of correction and to a fine. The court refrained from ordering preventive detention because it expected that Hoinka would not commit any new crimes being under the influence of his wife. According to his own statements, he had two illegitimate children from two different women, a daughter and a son^1^.

After his imprisonment, Hoinka settled in Cologne and earned his living as a sales driver for various beverage wholesalers. In 1960, he founded his own car rental business, as his earnings as a sales driver were not enough to meet his needs. He bought the necessary cars on credit. Later, in response to a marriage advertisement placed by him in the local newspaper, he met a businesswoman who became his partner based on Hoinka’s unrebutted promise of marriage. She participated in the business which was run under her name, and she appointed Hoinka as managing director. Despite her considerable financial support, however, the business collapsed after a short time and Hoinka separated from her at the beginning of 1961. The collapse of his business did not come as a surprise: Hoinka led a lavish and sophisticated lifestyle, dining at expensive restaurants, wearing tailored suits, staying in first-class hotels, and driving various expensive cars such as Jaguar, Mercedes-Benz, and Citroen. He persuaded at least 28 women to hand over cash, making promises of marriage with which, according to his own account, he wanted to either expand the business or pay off other partners. He also arranged for women to sign financing applications for the purchase of vehicles and bills of exchange. Hoinka met most of the women through marriage ads. In almost all cases, he promised them marriage and then took their savings, money they borrowed or their proceeds from the sale of property, supposedly to build a joint existence^2^. At his insistence or suggestion, most women allowed him to take nude photographs of them, confiding in the marriage promise made to them. Hoinka pretended that he wanted to carry the pictures with him as a remembrance of his future wife, since as a traveling businessman he would not be able to see her all the time. Since Hoinka used marriage advertisements, in order to avoid threatened disclosure, he first used the alias “Handke” and later “Markgraf.” One of his “brides” even placed these marriage ads for him. She made them in the name of Markgraf, not realizing that, in fact, they were for Hoinka himself^3^.

### 3.2 Psychiatric evaluations of Hoinka

The court in Cologne ordered psychiatric evaluations from two medical experts, Gerd Huber (1921–2012) and Fred Dubitscher (1905-1978)^3^. Gerd Huber was a clinical senior physician and extraordinary professor at the Psychiatric and Neurological Clinic at the University of Bonn. He had previously been an associate professor at the University of Heidelberg but moved to Bonn in 1962 at the invitation of Hans Jörg Weitbrecht (1909-1975), who was a professor of psychiatry and neurology at the University of Bonn. Huber specialized in schizophrenia research, but his early studies were considered controversial by his colleagues, as the neuropathological direction Huber took in psychiatry was new at the time and therefore met with criticism. It was not until the 1970s, with the development of better technical possibilities, that Huber’s findings were confirmed and acknowledged in the scientific community (8). However, Hoinka’s lawyer requested rejection of Huber’s expert opinion, accusing him of bias and prejudice in the evaluation^4^. Thus, Hoinka’s lawyer was able to arrange for a second medical expert, Fred Dubitscher. If Huber’s research was disputed because of its novelty, Dubitscher was an extremely controversial person, because he played a significant role in the research, dissemination, and practical implementation of Nazi racial policy (9). He was active as a racial hygienist from 1934 to 1945 for several institutions: the Department of Hereditary Medicine of the Reich Health Office, the Berlin Higher Hereditary Health Court, and the Higher Hereditary Health Court. Moreover, he was senior physician at the Polyclinic for Hereditary and Racial Care in Berlin-Charlottenburg. His activities included, among others, processing applications for sterilization, marriage loans, examinations on the question of marriage suitability and expert opinions for hereditary health courts (9). In 1942, Dubitscher published his book *Asoziale Sippen* (Asocial Families), in which he tried to create a basis for the legal sterilization of marginalized social groups (10). After the end of World War II, Dubitscher was banned from practicing by the American military government. He was denazified in 1949. After that, he began his work as a contract physician. In 1962, he was appointed government medical director of the State Office of Public Assistance of the State North Rhine. Dubitscher worked as a neurological expert, focusing on the treatment of brain-injured war victims.

Huber was able to convince the court of his impartiality. Also, Dubitscher confirmed Huber’s conclusions, so his opinion was accepted. Huber even invited Hoinka to participate in his lecture on social and forensic psychiatry in July 1964.

### 3.3 Gerd Huber’s evaluation

Since Hoinka had suffered a head injury in 1933, x-rays were taken which showed no change from earlier scans from 1952. Huber could not find any traumatic brain injury^5^. He also performed various psychological examinations: intelligence test, Rorschach test and sentence completion test. The Hamburg-Wechsler Intelligence Test showed an above-average intelligence level with an intelligence quotient of 115^6^. The Rorschach test contained ten inkblots, six of which Hoinka interpreted according to the classical meanings, the second he interpreted as a butterfly, the seventh as “fur half eaten by moths, damaged fur”, the tenth as “human skeleton, from the abdomen, pelvis, spine (pink/grey)”, and the ninth inkblot he could not interpret. Huber found Hoinka’s unusual sexual interpretation of the tenth inkblot to be the only peculiarity. However, since it was the only response of its kind, he could not draw any conclusions about the superiority of the sexual sphere. In Huber’s view, the Rorschach test was not very useful because Hoinka, in contrast to his usual lively talkativeness, limited himself to a minimum of answers with very general “vulgar interpretations.” Huber concluded that was obviously due to his suspicious caution and understandable fear that unfavorable conclusions regarding his personality structure could be drawn from the results of this projection test. In the Arntzen sentence completion test, Hoinka again gave meaningful completions befitting his intelligence, but in such general terms that little could be gleaned about his personality from them as well^2^.

According to Huber’s psychopathological analysis, the defendant appeared to be a psychopathic personality with pronounced pseudo-logistic traits (p. 206)^2^. Huber could detect neither a severe form of psychopathy nor a sexual desire disorder. The medical expert defined Hoinka’s personality structure as “unstable and attention-seeking” (*haltlos-geltungssüchtig)* (p. 206)^2^. Using Kurt Schneider’s typology of psychopathic personalities Huber’s description would combine such types as the “attention-seeking” (*geltungsbedürftig*), the “affectionless” (*gemütlos*) and the “weak willed” (*willenlose)* psychopath (11). Their primary characteristics were unsustainability, poverty of spirit, lack of a sense of guilt and remorse, and a craving for recognition (p. 196-197, 208-209, 226)^2^. According to Huber, Hoinka’s biography revealed an appalling lack of perseverance and consistency, not only in the professional but also in the personal sphere, where his inability to form a deeper bond glaringly stood out. He always wanted to appear to be more than he was. In addition, he presented a sentimental, inauthentic, theatrical image of himself and was full of self-pity, self-righteousness, and self-overestimation. It was typical for the inner attitude of the accused that he, with almost naive self-evidence, always claimed a standard of living for himself that presupposed an income that he could never achieve by honest means due to his lack of professional training. For this reason, the defendant never lasted long in his various jobs, but made himself self-employed, although he had neither the corresponding knowledge nor the necessary capital to do so. He blamed others for the difficulties that inevitably arose from this dichotomy between his ability and his will, his father who had not brought him up properly in his youth, his business partners who had taken advantage of him, or any adverse external circumstances. However, he never sought the reason for it in himself. He always unhesitatingly resorted to the only almost infallible means at his disposal to satisfy his excessive material demands: marriage swindling. In doing so, his good intellectual disposition, his apparent education and comprehensive knowledge of the female psyche acquired through growing up between three sisters enabled him to skillfully exploit human weaknesses. He lived “in his own theatre” and changed from one sexual object to another, partly without primary material interest as to solely satisfy his vanity, and partly with obvious material intentions.

Huber concluded that Hoinka did not suffer from a mental disorder, nor did he suffer from a mental weakness or a disturbance of consciousness. According to Huber, the defendant was fully capable of recognizing the wrongfulness of his actions. Regarding the social and criminological prognosis for Hoinka, Huber was unable to give a reliable assessment. However, due to Hoinka’s personality structure, there was a high probability that he would commit similar offences again in the future. Hoinka repeatedly committed the same offence within a very short period after his release from prison. Thus, the medical expert considered the offender inclined to commit further crimes (*Hangtäterschaft*) (p. 232)^2^. Huber did not believe in the improvement of Hoinka’s prognosis with increasing age and conceded that the defendant would have continued his activities even at an advanced age. Also, Hoinka’s pseudology could hardly improve in his life course, because according to Emil Kraepelin’s (1856-1926) studies there was just little chance of improvement after the age of 35. Given Hoinka’s characterological structure, Huber found him unsuitable for any psychotherapeutic treatment.

In his medical opinion, Huber also mentions that knowledge of Hoinka’s genealogical situation could be helpful in formulating a prognosis. This could become the case if a relative could be found who had a similar or identical personality structure (p. 233)^2^. Among the materials on Hoinka’s case, we found a paper entitled “On the question of the attention-seeking-pseudologist-hysterical personality (Hoinka case)” (*Zur Frage der geltungssüchtig-pseudologen-hysterischen Persönlichkeit, Fall Hoinka*), in which Huber wrote quite clearly: “an approximately reliable prognosis can only be made for a psychopathic personality if, in addition to its ‘individual general structure’, its genealogical situation is precisely known (if after extensive research into the kinship [*Sippe*], one comes across a relative who was just like that)” (p. 3)^7^.

### 3.4 Fred Dubitscher’s evaluation

Dubitcher was appointed as a second expert by Hoinka’s lawyer after Huber had been accused of being biased. However, Dubitcher described Huber’s expert opinion as complete, professional, and seamless^8^ Like his colleague, Dubitscher also concluded that, at the time of the crimes, the defendant had neither a disorder of consciousness, nor a pathological disturbance of mental activity, nor even a weakness of mind. He agreed with Huber that a social and criminological prognosis was not possible. However, psychotherapy was not seen as a proper intervention capable of changing Hoinka’s character structure. Dubitscher was surprised to find that Hoinka’s family history was inconspicuous given his far-reaching personality deviation. Yet, one would still have to trace back one or two generations. Dubitscher referred to his monograph *Asoziale Sippen* and emphasized that conspicuous features could be detected in the history of socially deviant people. Dubitscher went so far as to conclude that if this was not the case in Hoinka’s genealogy, the prognosis could still be more favorable given an inconspicuous “genetical structure” (*erbbiologische Struktur*) (p. 5)^7^. Dubitscher visited Hoinka in 1965 in prison in Cologne to get a personal impression and to discuss the family history of the defendant. However, the expert could not discover any peculiarities that could constitute some “genetical” evidence. It is interesting that Dubitscher pointed out that Hoinka did not commit any offence against the “general public” per se, but only against a particular group of women (p. 6)^7^.

Dubitscher agreed with Huber that new crimes of the same kind could be expected if Hoinka was to be left to his own devices. However, in his expert opinion, Dubitscher did not speak of preventive detention, but of resocialization. He suggested that Hoinka should receive an economically viable education, follow-up social assistance and employment after serving his sentence (p. 7)^7^. As he rightly pointed out, the defendant had not received any help of this kind so far.

### 3.5 Hoinka’s fate after the trial

The defendant Helmut Hoinka was sentenced as a dangerous habitual criminal for continued recidivist fraud in ten cases, recidivist fraud in eight cases, one of which was committed in combination with forgery of documents, attempted recidivist fraud in two cases, one of which was committed in combination with forgery of documents, to a total sentence of six years’ correctional imprisonment and a fine of 3,540 Deutsche Mark. Based on the convincing arguments of the medical experts Huber and Dubitscher, with which the Chamber fully agreed, there was an overwhelming probability that further similar crimes were to be expected after serving of the sentence. Thus, in addition to the sentence, preventive detention was ordered in accordance with § 42e of the Criminal Code, as it was deemed necessary for public safety^1^.

At the beginning of 1968, Hoinka lodged a complaint against the decision of the Cologne Regional Court and submitted a request for conditional release from imprisonment and suspension of preventive detention. That appeal was rejected as unfounded. The refusal was mainly based on the negative criminological prognosis made by medical experts^9^. After serving his sentence in 1970, Hoinka applied for conditional release from preventive detention, which this time was granted. The following conditions were imposed on him: to lead a crime-free life, to complete an internship in bookbinding, supervision by a probation officer, and not to change his residence or place of work without the court’s approval. He was ordered to take up residence with his bride in Würzburg^10^. The bride turned out to be one of the women who had been deceived by Hoinka and who had helped him write marriage advertisements. In 1974, after the expiration of the probationary period, the sentence was remitted out of mercy. At that time, Hoinka was 61 years old.

Interestingly, at the end of 1965, East German filmmaker Jürgen Sehmisch made the television movie *Der Rosenkavalier* (The Rose-Bearer) as an episode of the highly popular film series *Der Staatsanwalt hat das Wort* (The Prosecutor Has the Word). Although we do not have exact information on this matter, we can still suppose that news of Hoinka’s trial, who was also well known in post-war East Germany as a marriage swindler, was the inspiration for the movie.

## 4 Discussion

### 4.1 Gerd Huber’s evaluation of Hoinka

Gerd Huber submitted a very extensive and detailed expert opinion concerning Helmuth Hoinka on July 2^nd^, 1964. The question to be answered was whether Hoinka could be held criminally responsible for his offences and whether he would tend to commit similar acts again. Although Huber wrote his expert opinion in a technically flawless manner and meticulously compiled Hoinka’s background, a certain tendency and bias is noticeable, as well as some presupposed and unspoken moral values, which upon closer examination are by no means unproblematic. For example, the detailed psychopathological findings alone, which cover more than eight pages, are highly prejudicial. Hoinka is not treated well in the truest sense of the word. Huber mentions that Hoinka had a “clearly evident tendency to present as favorable a picture of himself as possible” (p. 186)^2^, which is why it was “not readily possible” to gain “a reliable, comprehensive and closed picture of his life story and his personality structure” (p. 186)^2^. Furthermore, Hoinka had “the ability [ … ] to live entirely in his own theatre [ … ], within which the appearance of genuineness is more or less compelling for the respective partner” (p. 188)^2^. Huber thus makes it clear that Hoinka was in no way considered credible by him, from which it follows that his own opinions of Hoinka should weigh more heavily than those of Hoinka himself. The suspicion of a certain prejudice also arises from the frequent use of subjectivist, derogatory words in relation to Hoinka, which after all call into question the neutrality and impartiality of the psychiatric report. Thus, Hoinka’s life is described as “botched” (p. 187)^2^, Hoinka’s methods as “smug exaggeration, cutting and boasting, lies and swindles” (p. 188)^2^, and much more. Also, in the entire detailed justification of his expert opinion, Huber uses almost exclusively pejorative descriptions to describe Hoinka’s character and deeds. Only in rare cases does Huber use a positive attribution, for example when he describes Hoinka’s arousal of sympathy in women as “skillful” (p. 212)^2^ or when his clothing is described as distinguished by “captivat[ing] elegance and fashionable correctness” (p. 217)^2^. In these rare cases, however, one has less of the impression that Huber really wants to mention a positive aspect of Hoinka, but rather that he wants to underline the reprehensibility of his behavior. Interestingly, Huber also lets his own moral ideas come through, which find their way into the evaluation of Hoinka. In his words, Hoinka’s character structure could be defined by a “lack of any determination and perseverance” (p. 204)^2^. Hoinka had always “avoided all inconveniences” (p. 205)^2^ and was “not prepared to take the arduous path of advancement” (p. 205)^2^. Hoinka had “always aimed at achieving a maximum of enjoyment, pleasure and comfort with a minimum of effort” (p. 207)^2^. These formulations may not only inform us about Hoinka’s character, but rather reveal Huber’s values, who obviously seems to regard strenuous work as a prerequisite for success and thus may be biased to some extent in relation to Hoinka’s life. Huber’s comments and evaluations of Hoinka’s sexuality are also not free of subjectivity. They reveal an attitude that, although possibly due to the times in which his expert opinion was written, is at least in part rather conservative. This could have influenced Huber’s neutrality in respect to Hoinka. Huber condemns the objectification of the sexual partner and sees sexuality as “rightly [ … ] based on ‘we-formation’, on genuine partnership that transcends the sexual sphere” (p. 224)^2^. Hoinka, like other people, had shown “a failure in the face of the individual task” (p. 225)^2^, “to resist the inclination coupled with the libido to live out a sexuality that has been denuded of Eros and has become impersonal” (p. 225f)^2^. There are also very tendentious descriptions of the nude photographs found in Hoinka’s documents. In their “hardly surpassable tasteless primitiveness,” they showed that the women depicted were only sexual objects (p. 231)^2^. In addition to the very pejorative attitude towards pornographic image material in general, Huber also denies, at least to some extent, that women may also sometimes, although certainly not exclusively, wish to be seen as sexual objects. Here again, Huber seems to be describing rather personal and subjective attitudes that at the very least call the neutrality of the expert into question. All in all, there is no attempt in his entire expert report to interpret Hoinka’s behavior in any other way than as stemming from a basic need for recognition. One would have wished for a more differentiated presentation from Huber that at least tried to understand Hoinka’s perspective. Huber finally denies Hoinka such an understanding himself when he declares him “unsuitable for any treatment of a psychotherapeutic nature” (p. 233)^2^.

### 4.2 Dubitscher’s evaluation

The first thing that draws attention in Dubitcher’s expert opinion is that he wrote openly about his book *Asoziale Sippen* and supported the “genetic-biological” approach. The mere use of the terms “*Erbbiologie*”, “*Asoziale*” and “*Sippe*” from the Nazi period was already unacceptable. Surprisingly, no one in the court or the defendant’s defense drew attention to his use of Nazi terminology or to his appeal to racist theory. In his book *Asoziale Sippen* (10), Dubitscher had described various families and their members with an illustration of their family tree. His book can thus be seen as a “graphic implementation of the racial doctrine of the time” (9, p. 131). According to his topology of “asocial persons,” he distinguished work-shy or unemployed, beggars, tramps, and vagabonds, “morally depraved,” “antisocial criminals”, and others. Although Dubitscher’s research gave no evidence of the heritability of “antisocial characteristics,” he demanded the sterilization of “asocial” persons. As we see in his opinion, he attempts to clarify Hoinka’s genealogy and only because he finds no unusual cases in the family of the defendant, Dubitscher concludes that the prognosis could be positive. In this respect, we can agree with Dubitscher’s biographers that his belief in inherited mental and moral qualities by no means ended with the Nazi rule, but continued after 1945 (9, p. 146). Following the typology of his book, Hoinka could be classified as an “antisocial criminal” who, in Nazi times, would have faced sterilization or even “extermination through labor.” However, Dubitscher, unlike Huber, proposes resocialization of the defendant and the creation of favorable conditions for him, under which he would not find himself wishing to return to crime. However, it remains unknown whether he truly believed so and whether it mattered that he was summoned as an expert by the defendant’s defense, and not by the prosecution.

### 4.3 Role of the psychiatrists as medical experts

Before imposing preventive detention, the judge must assess whether the defendant will be likely to reoffend in the future and how dangerous he or she will be to society. Obviously, the judge is not in a position to answer these questions. Therefore, the support of medical experts is necessary. They can give an expert opinion on the health and further dangerousness of a defendant. The difficulties in assessing the dangerousness of the offender and providing a prognosis on further delinquency remain the focus of discussions about preventive detention to this day (4, 12–15). As legal scholars have pointed out, medical experts cannot be assumed unprejudiced, as they already are aware of the defendant’s previous convictions prior to the assessment, which might be a source of bias (4, p. 191). Furthermore, one should take into account that the criteria for the assessment of the delinquent as a dangerous habitual offender allowed for a wide range of interpretation. Thus, both a very low and a high IQ could be interpreted in disfavor of the defendant (4, p. 191). In Hoinka’s case, as we could show, his high level of intelligence was constantly emphasized by Huber in a negative way. The medical expert believed that neither the Rorschach test nor the Arntzen sentence completion test could be considered reliable, because Hoinka might have manipulated his answers. Moreover, as we have shown in our analysis of Huber’s opinion, he allowed himself personal value judgments in assessing Hoinka’s personality. This might be due to the fact that the definition of psychopathy used at the time was not neutral, but normative and judgmental (16). Even Kurt Schneider, being a critic of Kraepelin’s concept of the “psychopathic personality” for including moral criteria, repeated the stand of his predecessor in attempting to compile objective criteria. In describing the “affectionless” (*gemütlos*) psychopathic type, Schneider included such criteria as a lack of “pity, shame, honor, repentance, conscience” (11). However, an individual’s deviant or criminal behavior cannot be considered a medical symptom of psychopathy merely because it violates common moral and social norms (17). The focus on affective traits in defining psychopathy, which was especially relevant in German psychiatric tradition, was abandoned in 1980 with the publication of the Third Edition of the Diagnostic and Statistical Manual of Mental Disorders (18). For the first time, psychopathy was defined from a behavioral standpoint as the persistent violation of social norms. However, the label “antisocial personality disorder” introduced in 1952 remained (16). Despite the difficulties in establishing reliable criteria for psychopathy, in the 1960s a diagnostic and treatment manual regarding psychopathy for forensic use still did not exist (16). It was published only in 1980, and included both affective and behavioral factors (19).

The medical reports of both experts showed that the psychiatrists believed in a genetic nature of psychopathy and criminal behavior. As we have seen above, the criminological prognosis of both medical experts was fully supported by the court in imposing the sentence. This same prognosis was also relied upon by the court in 1968 when it denied Hoinka’s request for conditional release from the house of correction and revocation of preventive detention.

Since then, there has been a paradigm shift in forensic psychiatry. It is no longer a matter of criminological prognosis, but of risk assessment. If prognosis is the prediction of what will happen, risk assessment is the determination of what must be changed in order to prevent a danger from becoming a reality. Moreover, forensic psychiatrists are constantly refining mechanisms for evaluating offenders as well as prognostic assessment tools. Intelligence test and personality tests, such as the Minnesota Multiphasic Personality Inventory (MMPI-2), are used to measure individual abilities, skills and personality of offenders. In the area of personality measurement, the MMPI-2 also offers differentiated possibilities for assessing falsification tendencies. Not only psychological tests, but also modern imaging techniques help to diagnose personality disorders. Therefore, looking at the historical case, it is difficult for us to say what conclusion psychiatrists would have made nowadays when they evaluated Hoinka.

## 5 Conclusion

As we have seen in Hoinka’s case, the opinion of medical experts was crucial in imposing his sentence. They not only evaluated his criminal responsibility, but also the possibility of recidivism in the future. Even though neither expert could give a clear social and criminological prognosis for Hoinka, they still assumed that the defendant would return to crime after serving his sentence. The assessment of the dangerousness of the offender is still a complex issue to this day. In the 1960s, the challenge was compounded by the fact that there were neither clear criteria for antisocial personality disorder, then called psychopathy, nor a diagnostic or treatment manual regarding personality disorders for forensic use. All of this was compounded by the personal bias of medical experts and their belief in the theory of genetic predisposition to crime. Both the experts did not suggest psychotherapy, because Hoinka did not have any pathological disturbance of mental activity. Although Hoinka was declared a highly dangerous offender and sentenced to preventive detention, the latter was eventually abolished. This case shows that resocialization - the necessity to work and return from prison to family relations - has proved to be an effective preventive measure.

## Data availability statement

The original contributions presented in the study are included in the article/supplementary material. Further inquiries can be directed to the corresponding author.

## Ethics statement

Written informed consent was not obtained from the individual(s) for the publication of any potentially identifiable images or data included in this article because it is a historical case. We used a photo of Helmut Hoinka that had already been published in 1965.

## Author contributions

OK: Conceptualization, Formal analysis, Investigation, Methodology, Resources, Validation, Writing – original draft, Writing – review & editing. TS-E: Conceptualization, Formal analysis, Investigation, Methodology, Validation, Writing – original draft, Writing – review & editing. FS: Conceptualization, Formal analysis, Investigation, Methodology, Resources, Validation, Writing – review & editing.

## Footnotes

1 Verdict of the 3rd Grand Criminal Chamber of the Regional Court in Cologne, 29.07.1965. Landesarchiv NRW, Abt. Rheinland. Gerichte Rep. 287 Nr. Hoinka, Helmut (1933).

2 Huber G. Scientific report on mr. Helmuth Hoinka, 02.07.1964. Gerd Huber Archive.

3 The main hearings of the 3rd Grand Criminal Chamber of the Regional Court in Cologne on July 19, 20, 23, 26, 27 and 29, 1965. Landesarchiv NRW, Abt. Rheinland. Gerichte Rep. 287 Nr. Hoinka, Helmut (1933).

4 Ungar H. Letter to the Regional Court of Cologne, 05.10.1964. Gerd Huber Archive.

5 Huber G. Oral expert opinion. 27.07.1965. Gerd Huber Archive.

6 Hoinka H. Hamburg-Wechsler intelligence test for adults, 21.07.1964. Gerd Huber Archive.

7 Huber G. Zur Frage der geltungssüchtig-pseudologen-hysterischen Persönlichkeit, Fall Hoinka, 1965. Gerd Huber Archive.

8 Dubitscher F. Expert opinion on Helmut Hoinka, 17.05.1965. Gerd Huber Archive.

9 Decision in the criminal case against the driver Helmut Rudolf Adolf Hoinka. 22.03.1968. Landesarchiv NRW, Abt. Rheinland. Gerichte Rep. 287 Nr. Hoinka, Helmut (1933).

10 Decision in the criminal case against Hoinka, 20.07.1970. Landesarchiv NRW, Abt. Hoinka, Helmut: Rheinland. Gerichte Rep. 287 Nr (1933).
